# Prevalence and associated risk factors of intestinal parasitic infections among asymptomatic food handlers in Wollo University student’s cafeteria, Northeastern Ethiopia

**DOI:** 10.1186/s13104-019-4182-7

**Published:** 2019-03-14

**Authors:** Edosa Kebede, Abdurahaman Seid, Senayt Akele

**Affiliations:** 1 Department of Medical Laboratory Sciences, College of Medicine and Health Sciences, Wollo University, Dessie, Ethiopia; 2 Department of Medical Laboratory Sciences, Students Clinic, Wollo University, Dessie, Ethiopia

**Keywords:** Intestinal parasites, Food handlers, Prevalence, Ethiopia

## Abstract

**Objective:**

Intestinal parasitic infections (IPIs) are among the major public health and socioeconomic problems in developing countries like Ethiopia. Food-handlers that directly deal with production and distribution of foods between societies are one of the most important sources to transmit parasitic infections to humans. The aim of this study was to assess the prevalence and associated risk factors of intestinal parasites among asymptomatic food-handlers working in students’ cafeteria of Wollo University, Northeastern Ethiopia.

**Result:**

A cross-sectional study was conducted among 200 asymptomatic food handlers at Wollo University from January 1 to February 20, 2018. Among the total participants 30 (15%) were infected with at least one intestinal parasites. The dominant parasite was *E. histolytica* (5.5%) followed by *A. lumbricoides* (4%) and then *G. lamblia* (3%). Finger nail trimming (p = 0.002, AOR: 4.35, 95% CI 1.71–11.04), medical checkup (p = 0.012, AOR: 4.01, 95% CI 1.37–12.25) and residence (p = 0.014, AOR: 3.16, 95% CI 1.26–7.95) were independent predictors of intestinal parasitic infection among the food handlers.

## Introduction

Food borne diseases are public health problems worldwide. The overall estimated parasitic food borne disease was 357 million cases and 33,900 deaths in 2010 [1].

Intestinal parasitic infection is one of the major health issues globally and assessed to infect about one third of the total population, the larger part being individuals living in tropical and sub-tropical parts of the world [2].

Ethiopia features a higher burden of IPIs and positioned the moment most noteworthy burden of ascariasis, the third most noteworthy burden of *hookworm* and the fourth highest burden of trichuriasis in Sub-Saharan Africa [3]. For this reason, the Ethiopian government considers intestinal parasite infection as one of the needs among neglected tropical diseases control programme [4].

Many research works indicated different factors such as lack of personal and environmental sanitation, lack of safe water supply, human behavior, poverty, and ignorance of health promotion practices had association with prevalence of IPIs among food handlers [5–7].

Food handlers with poor personal hygiene in food establishment can be considered as dangerous to the society because they are unaware of their potential to transmit [8, 9]. Additionally, food establishment centers such as University cafeterias prepare food in large amount which increases the chance of contamination once food handlers do not maintain proper sanitary practice [10]. Food contamination may happen at any steps and the healthy person (consumers) may be infected by eating or drinking this contaminated foodstuff [11].

According to several researches done on food handlers [5–7, 12, 13] the prevalence of IPIs in different parts of Ethiopia was found to be 25–49%. Due to this, periodical assessment of the prevalence for future mediation in risk group is very important. In addition, Ethiopian universities including Wollo University are experiencing speedy increase in student intake potential and cafeteria centers as well. For this reason, guaranteeing hygienic practices of food handlers is extremely vital to protect the health of the students. In addition, information regarding prevalence and associated risk factors of IPIs among food handlers is limited in the study area. Therefore, the aim of this study was to assess the prevalence of IPIs and associated factors among asymptomatic food handlers working in the Wollo University student cafeteria.

## Main text

### Methods

#### Study design, period and area

A cross-sectional study was conducted among asymptomatic food handlers at Wollo University from January 1 to February 20, 2018. Wollo University is found in Dessie town, northeastern Ethiopia in Amhara National Regional State, South Wollo zone at a distance of 401 Km from Addis Ababa, the capital city of Ethiopia. Its astronomical location is 11°8′N latitude and 39°38′E longitude.

#### Study population and sampling technique

Study population comprised of asymptomatic food handlers working in Wollo University cafeterias. About 500 food handlers were working in Wollo university students’ cafeteria during the study period and their list was obtained from human resource management of Wollo University and a simple random sampling technique using the lottery method was used to select the study subjects.

#### Inclusion and exclusion criteria

All food handlers who were working at Wollo University cafeteria at the time of study were included in this study and those who have taken anti parasitic drugs in the last 3 weeks before the study were excluded.

#### Sample size determination

The sample size was determined using sample size determination for estimation of single population proportion formula and calculated by assuming a previous prevalence of 25% [14], 95% confidence interval and 5% marginal error (d = 0.05).

$${\text{n}} = \frac{{\left( {{\text{Z}}_{{\upalpha/2}} } \right)^{2} * {\text{P}}\left( {1 - {\text{P}}} \right)}}{{{\text{d}}^{2} }} = 288$$

Since the total number of the source population was 500 and below 10,000 a correction formula was used as follows:

$$\frac{n}{1 + n/N} = \frac{288}{1 + 288/500} \approx 1 8 2$$

By considering a 10% (≈ 18 subjects) non response rate, the final sample size became 200.

#### Specimen collection and examination

Socio-demographic data, and other associated risk factors were collected using interview by pre tested structured questionnaire. An estimated pea size fresh stool sample (2–3 g) was collected in a labeled, clean wide-mouthed plastic container. After receiving of the specimen, microscopic examination of the stool sample was done by direct wet mount preparations in normal saline and iodine solution. Formol ether concentration sedimentation techniques was also done as per the standards [15].

#### Data quality management

Microscopic examinations were done by experienced laboratory technologist with at least 2 years work experience. Cross checking of slides was done blindly by other laboratory technologist and a standard operational procedure of examinations was followed. Careful cleaning, coding and entering of data were done.

#### Ethical considerations

The study was ethically approved by a review committee of the Department of Medical Laboratory Sciences of Wollo University. A written informed consent was obtained from all study participants before data collection. Participants who have found to be positive for intestinal parasite were treated.

#### Data analysis

Epi-info version 3.5.1 was used to enter the data and it was transferred to SPSS version 20 software for analysis. The binary logistic regression model was used to assess the association between independent variables and IPIs. Variables that had a *p *<0.3 in the bivariate logistic analysis were run in multivariable logistic regression at 95% CI to observe their effect on the dependent variable by controlling possible confounding factors. A *p *<0.05 was taken as statistically significant.

### Result

#### Socio demographic characteristics

A total of 200 food handlers were participated in this study (145 females and 55 males). The age range of the study participants was from 18 to 52 with a mean age of 30.1 (SD ± 7.2). The majority (43.0%) lay in the age group of 21–30 years. The residence of study participants showed that 75.0% of them were urban dwellers. The majority of the study participants had completed secondary school 108 (54.0%) and 118 (59.0%) had work experience of 1–5 years (Table 1).

**Table 1:** Socio-demographic characteristics of food handlers in Wollo University student’s cafeteria, 2018 (N = 200)

Variables	Frequency	Percentage
Sex		
Male	55	27.5
Female	145	72.5
Age (in years)		
≤ 20	24	12
21–30	86	43
31–40	70	35
> 40	20	10
Residence		
Rural	50	25
Urban	150	75
Educational status		
Illiterate	19	9.5
Primary school	35	17.5
Secondary school	108	54
Higher	38	19
Service (in year)		
< 1	47	23.5
1–5	118	59
> 5	35	17.5

#### Prevalence of intestinal parasites

From the total of 200 stool specimens, 15.0% were positive for at least one parasite species. Five types of intestinal parasites were identified. The most prevalent parasite was *E. histolytica* (5.5%) followed by *A. lumbricoides* (4.0%) and then *G. lamblia* (3.0%) (Table 2).

**Table 2:** Identified intestinal parasites among food handlers in Wollo University student’s cafeteria, 2018 (N = 200)

Type of parasite isolates	Frequency	Percentage
*E. histolytica*	11/200	5.5
*A. lumbricoides*	8/200	4
*G. lamblia*	6/200	3
*Taenia species*	3/200	1.5

#### Factors associated with intestinal parasitic infections

Several factors were assessed for possible association with IPI among the food handlers as shown in Table 3. The study indicated individuals who lived in rural were 3 times (p = 0.014, AOR: 3.16, 95% CI 1.26–7.95) more likely to be infected with intestinal parasites than those who lived in urban. Similarly, the odds of parasitic infection was 4 times more likely (p = 0.012, AOR: 4.01, 95% CI 1.37–12.25) for individuals who had no medical checkup in the last 6 months as compared who did. In this study, untrimmed fingernails showed a higher prevalence than the trimmed ones and the difference was statistically significant (p = 0.002, AOR: 4.35, 95% CI 1.71–11.04). However, the association was not statistically significant for sex, age, educational status, service in year, food safety training, hand washing after toilet with soap and hand washing before food preparation with soap (Table 3).

**Table 3:** Logistic regression analysis of factors associated with IPIs among food handlers in Wollo University student’s cafeteria, 2018 (N = 200)

Variables	Infected (%)	COR (95% CI)	AOR (95% CI)	P-value
Sex				
Male	7	1.29 (0.52, 3.21)	–	–
Female	23	1		
Age (in years)				
< 20	5	0.67 (0.14, 3.24)	–	–
21–30	12	1.01 (0.28, 4.29)		
31–40	10	1.06 (0.26, 4.29)		
> 40	3	1		
Residence				
Rural	15	3.86 (1.72, 8.64)	3.16 (1.26, 7.95)	0.014*
Urban	15	1	1	
Educational status				
Illiterate	6	0.19 (0.04, 0.86)	–	–
Primary school	8	0.29 (0.07, 1.20)	–	–
Secondary school	13	0.63 (0.17, 2.33)	–	–
Higher	3	1	–	–
Service (in year)				
< 1	7	0.54 (0.13, 2.24)	–	–
1–5	20	0.46 (0.13, 1.65)		
> 5	3	1		
Medical checkup (in the last 6 months)				
No	25	4.34 (1.59, 11.88)	4.01 (1.37, 12.25)	0.012*
Yes	5	1	1	
Food safety training				
No	25	1.97 (0.71, 5.44)	–	–
Yes	5	1	–	–
Finger nail trimming				
No	16	5.12 (2.27, 11.59)	4.35 (1.71, 11.04)	0.002*
Yes	14	1	1	
Hand washing after toilet with a soap				
No	10	1.01 (0.44, 2.3)	–	–
Yes	20	1		
Hand washing before food preparing with a soap				
No	8	1.63 (0.66, 4.00)	–	–
Yes	22	1		

*COR* crude odds ratio, *AOR* adjusted odds ratio, *CI* confidence interval

*p < 0.05

### Discussion

The result of this study showed that 15.0% of the food handlers were infected with intestinal parasites. The finding was similar with studies from different parts of Ethiopia such as Aksum town 14.5% [16], Bahirdar University 12.9% [17], and other countries, such as India and Iran, display similar rates of infection [18–21]. However, it is relatively higher than the 6.9% and 3.73% reported from Sudan and Iran respectively [8, 22]. But this finding is lower than the findings of other studies [5, 6, 14, 23–26] which reported 25.2%, 33.0%, 25.0%, 33.68%, 29.1%, 36% and 29.4% respectively. It was much lower than the studies from the other parts of Ethiopia [7, 12, 13] which reported 49.4%, 45.3%, 41.1% respectively and different countries such as Nigeria and Pakistan [27, 28] displayed 41.2% and 83.1% prevalence. The variation in reported prevalence in various studies might be due to socio-demographic features, personal hygiene, time of the study and geographical variation of the participants.

The predominant parasite identified in the present study was *E. histolytica* with a prevalence of 5.5% followed by *A. lumbricoides* (4.0%). This was in line with the finding of similar studies conducted in different parts of Ethiopia [5, 7, 17], in which *E. histolytica* was the predominant parasite reported with a prevalence of 46.7%, 36.6% and 12.7% respectively. However, the predominant organism was found to be *A. lumbricoides* in other studies [6, 23, 24]. The variation could be due to variable food habits, cultural factors and geographical conditions.

A common practice which results in food borne illnesses among food handlers is inappropriate hygienic practices, particularly poor hand washing [29].

Respondents hand washing practice after using the toilet was (66.5%) which is in line with the report from Mekelle 70.4% [7] but lower than study conducted in Bahirdar 90.6% [13]. However, it is higher than study conducted in Jimma 48.9% [6]. The variation could be due to educational status, inadequate sanitary surveillance by regulatory team and scarcity of hand washing facilities in a working environment.

In this study there was no statistically significant association between the intestinal parasitic infection and age, sex, service year, hand washing habit with soap after toilet, hand washing with soap before food preparation, food safety training and educational status which is in line with other studies in the country [13, 23]. The discrepancy might be the small sample size and social desirability bias, in particular for hand washing practices.

In this study, 42.0% of the respondents had a medical checkup in the last 6 months. This is relatively similar with a report from Mekelle [7], which is 36.8%, but higher than 14.5% reported by Gezehegn et al. [16] and no medical checkup in a study from Bahirdar town [13]. This discrepancy may be due variation in educational status among participants and standard supervision by administrative group.

The odds IPIs was four fold higher among food handler who had no medical checkup in the last 6 months compared to those who had which was in agreement with another study from Ethiopia [17].

Higher prevalence (30.0%) of intestinal parasitic infection was seen in rural areas than urban (10.0%) and it also showed statistically significant association. Similar results were reported by others [7, 18]. This could be due to poor awareness about hygienic practices and lesser availability of clean water.

Food handlers who did not trim their fingernails had four times higher odds having IPIs compared to food handlers who did. Similar findings were reported by others [5, 16, 30]. This could be due to the area underneath untrimmed finger nails is troublesome to clean and harbors most organisms.

### Conclusion

In conclusion, the prevalence of IPIs among food handlers in this study was 30 (15%). The study also identified not trimming finger nail, not having regular medical checkups and being rural as risk factors of intestinal parasitic infection. For this reason, periodic screening of food handlers for parasites and health education on an appropriate trimming or cleaning of fingernails should be fortified for prevention of food borne illnesses.

## Limitations

In the present study carriage of the finger nail contents was not assessed.

Another concern is social desirability bias, which might cause weak association of hand washing practices with IPIs.

In this study, if combination of methods and multiple stool samples had been used, much greater rates of parasites would have been recovered.

## Abbreviation

IPIs: intestinal parasitic infections
